# Learning behavior, student engagement, and academic performance in learning analytics

**DOI:** 10.3389/fpsyg.2026.1908197

**Published:** 2026-09-14

**Authors:** Wenwei Huang, Cui Yue

**Affiliations:** 1 School of Marxism, HeNan Geology Mineral College, Zhengzhou, Henan, China; 2 School of Marxism, Xuchang University, Xuchang, Henan, China

**Keywords:** academic performance, educational measurement, learning analytics, learning behavior, predictive modeling, student engagement

## Abstract

**Introduction:**

Learning behavior and student engagement are important educational constructs that are associated with academic performance. Learning analytics provides opportunities to examine these relationships using large educational datasets. However, clear construct separation, appropriate statistical validation, and comparison with alternative predictive models remain necessary.

**Methods:**

We analyzed a publicly available educational analytics dataset containing 14,003 records. After removing 1,534 exact duplicate records, we retained 12,469 unique records. Learning behavior was represented by study hours, online course participation, and assignment completion. Student engagement was represented by attendance, discussion participation, and extracurricular involvement. Academic performance was represented by examination score, while final grade was excluded because its calculation procedure was not documented. We used ordinary least squares regression to examine the statistical associations. We also included an observed variable path model and machine learning models as alternative specifications. We used an 80% training and 20% held-out test split for predictive validation.

**Results:**

Learning behavior showed a small and nonsignificant association with student engagement (β₁ = 0.0017, *p* = 0.8112). Student engagement showed a negative association with academic performance (γ₁ = −1.2485, *p* = 0.0014), whereas learning behavior showed a positive association (γ₂ = 1.0075, *p* = 0.0009). The baseline model showed limited explanatory power (R^2^ = 0.004). On the held-out test set, the random forest model provided the strongest predictive performance (R^2^ = 0.564, RMSE = 11.719, MAE = 8.896, and MAPE = 14.013%). The robustness analysis showed similar coefficient magnitudes across the alternative static specifications.

**Discussion:**

The findings indicate statistical associations among learning behavior, student engagement, and academic performance within the analytical dataset. They do not establish causal relationships or temporal relationships because the dataset is cross-sectional and contains no documented temporal ordering or repeated observations. The results support transparent construct definition, comparative model evaluation, and out-of-sample validation in learning analytics research.

## 1 Introduction

Academic performance is an important indicator of educational achievement, but its interpretation requires attention to the behavioral and contextual factors that accompany learning (Costa et al., 2024; Aarthi et al., 2026). Student engagement is one of the most established constructs in this area. It is commonly represented through behavioral, emotional, and cognitive dimensions, and its measurement has been associated with academic achievement, persistence, and other educational outcomes (Fredricks et al., 2004; Tu et al., 2026). Learning behavior provides a related perspective because study effort, participation, assignment completion, and learning practices provide observable indicators of how students interact with educational activities (Zhu et al., 2026; Wu et al., 2026).

The development of learning analytics has created new opportunities to study these relationships (Shi et al., 2025; Tang et al., 2026). Learning analytics uses data about learners and their educational contexts to examine learning processes and educational outcomes (Siemens, 2013; Luo et al., 2023). Educational data mining provides related computational methods for identifying patterns and making predictions from educational data, while closer communication between learning analytics and educational data mining has been proposed as a means of improving methodological development in the field (Siemens and Baker, 2012; Zhou et al., 2021). Recent studies have therefore examined attendance, online activity, participation, assessment records, and other behavioral indicators as predictors of student outcomes.

A methodological issue arises when several observed indicators are combined to represent broader educational constructs. Composite measures require a clear theoretical definition of the construct, appropriate indicator assignment, and careful interpretation of the resulting scores. Educational measurement research emphasizes that the validity of an inference depends on the evidence and theoretical rationale that support the interpretation of the score (Messick, 1989). This consideration is particularly relevant when learning behavior, engagement, and academic performance are represented by indices derived from different observed variables.

A second issue concerns the distinction between cross sectional association and longitudinal development. Educational development may be studied through repeated observations when the research question concerns change over time, individual trajectories, or accumulated experience. Longitudinal methods such as latent growth curve models are specifically designed for repeated measurements and the analysis of change over time (Duncan et al., 2013). In contrast, the dataset used in the present study contains cross-sectional records without documented temporal identifiers or repeated observations. The present analysis therefore does not estimate individual developmental trajectories or temporal changes.

Longitudinal educational research can also examine temporal relationships when repeated observations and documented time ordering are available (Zhou et al., 2021; Li et al., 2025). Such data may support more advanced temporal models, including fractional order approaches. However, the present dataset is cross-sectional and contains no documented temporal identifiers or repeated observations. Therefore, fractional order modeling is outside the scope of the present empirical analysis. Such models may be relevant to educational research when longitudinal observations provide a genuine temporal sequence and when accumulated historical observations form part of the research question. However, the absence of a temporal variable in the present dataset does not permit direct empirical estimation of individual learning memory or temporal dependence.

The methodological gap this study addresses is therefore narrower than a claim of empirically established fractional memory. Existing educational research provides substantial evidence on student engagement, learning behavior, and academic performance, while learning analytics provides established methods for extracting predictive information from educational records. Less attention has been given to a unified statistical representation of these constructs within a transparent cross-sectional framework that separates construct definition, statistical association, model comparison, and predictive validation. This study addresses this gap by constructing nonoverlapping indices for learning behavior, student engagement, and academic performance and evaluating their statistical relationships using conventional regression and predictive benchmarks.

The study has three objectives. First, it examines the statistical association between learning behavior and student engagement. Second, it evaluates the association between student engagement, learning behavior, and academic performance after adjustment for selected individual and contextual variables. Third, it compares the baseline statistical specification with an observed variable path model and machine learning alternatives and examines the stability of the results under alternative static specifications.

The study’s contribution is therefore empirical and methodological. It provides a transparent cross-sectional analysis of educational indicators within a publicly available dataset, applies explicit construct definitions and validation procedures, and evaluates the resulting relationships against appropriate alternative models. Fractional calculus offers a potential mathematical approach for future longitudinal educational research with repeated observations and explicit temporal ordering. Because the present dataset is cross-sectional and contains no documented temporal identifiers or repeated observations, this study does not estimate fractional differential equations.

### 1.1 Literature Review

The relationship between student engagement and academic performance has been extensively examined within educational psychology and learning analytics research. Student engagement is commonly defined as the degree of behavioral, cognitive, and emotional involvement that students exhibit during the learning process (Papageorgiou et al., 2025; Uzun et al., 2025). Previous studies have consistently demonstrated that highly engaged students achieve superior academic outcomes, exhibit greater persistence, and report stronger learning satisfaction than their less engaged counterparts (Johar et al., 2023; Wang et al., 2025; Bergdahl et al., 2024).

Recent developments in learning analytics have enabled researchers to examine engagement through large-scale educational data (López-Meneses et al., 2025; Kaliisa et al., 2025). Learning analytics approaches utilize behavioral indicators such as attendance, assignment completion, online participation, and learning activity records to predict educational outcomes. Johar et al. (2023) reported that learning analytics significantly improves the understanding of student engagement patterns and provides valuable insights for enhancing academic performance. Similarly, Xu et al. (2025) demonstrated that behavioral learning indicators reliably predict student achievement across various educational settings.

The role of learning behavior has also received increasing attention in contemporary educational research. Learning behavior reflects the habits, practices, and strategies students adopt throughout the educational process. Studies have shown that effective learning behaviors, including consistent study effort, active participation, and self-regulated learning practices, contribute positively to academic achievement. Furthermore, motivation educational technology usage have been identified as factors that are associated with both engagement and performance (Al-Zahrani and Alasmari, 2023).

Although substantial progress has been achieved in understanding student engagement and academic performance, most existing studies rely on conventional statistical and machine learning techniques, including regression analysis, structural equation modeling, random forests, and neural networks. These approaches generally assume that educational outcomes depend primarily on current conditions and often do not explicitly represent the association between past learning experiences and current outcomes.

Knowledge acquisition occurs gradually, and current academic outcomes may correspond with learning experiences accumulated over extended periods. Although educational processes may involve changes over time, the present study is restricted to cross sectional data. The available records do not contain repeated observations or documented temporal identifiers. Therefore, the empirical analysis focuses on statistical associations and predictive relationships among learning behavior, student engagement, and academic performance.

### 1.2 Research Gap and Study Contributions

Three gaps are identified in the existing literature. First, student engagement and learning behavior are often examined through separate indicators or predictive models, making it difficult to compare relationships among constructs. Second, composite educational measures require explicit construct definitions and appropriate separation of indicators, but applied learning analytics studies do not always address this issue. Third, longitudinal and memory-based models require repeated observations and temporal ordering, whereas many available educational datasets are cross-sectional.

This study addresses these gaps through a cross-sectional analytical framework. Learning behavior, student engagement, and academic performance are represented by explicitly defined indices with nonoverlapping indicators. We estimate their statistical relationships using ordinary regression and compare them with structural and machine-learning alternatives. Robustness analysis tests whether the principal associations remain stable under alternative static specifications.

The contribution is therefore an empirical and methodological analysis of cross-sectional educational indicators. Future research may use longitudinal datasets with repeated observations to examine temporal relationships that cannot be evaluated with the present data. Future longitudinal studies with repeated observations and documented temporal ordering may provide an appropriate basis for examining temporal and fractional order models.

## 2 Methodology

### 2.1 Research design

This study adopts a quantitative cross-sectional research design to examine associations among learning behavior, student engagement, and academic performance. Because the dataset does not contain repeated measurements or a documented temporal variable, the analysis does not estimate temporal changes or dynamic relationships. The Learning Behavior Index, Student Engagement Index, and Academic Performance Index are therefore treated as cross sectional measures, and their statistical associations are examined within the available dataset. The analysis therefore treats the Learning Behavior Index, Student Engagement Index, and Academic Performance Index as cross-sectional measures and examines their statistical associations.

As illustrated in Figure 1, the proposed conceptual framework represents learning behavior, student engagement, and academic performance as related constructs. Learning behavior is represented through study hours, online course participation, and assignment completion. Student engagement is represented through attendance, discussion participation, and extracurricular involvement. Academic performance is represented by examination performance. The framework organizes the statistical associations among these constructs. Because the available dataset contains cross-sectional records without repeated measurements or documented temporal identifiers, the analysis does not establish temporal ordering or causal direction. Therefore, the estimated relationships are interpreted as statistical associations and predictive relationships within the analytical dataset.

**Figure 1:**
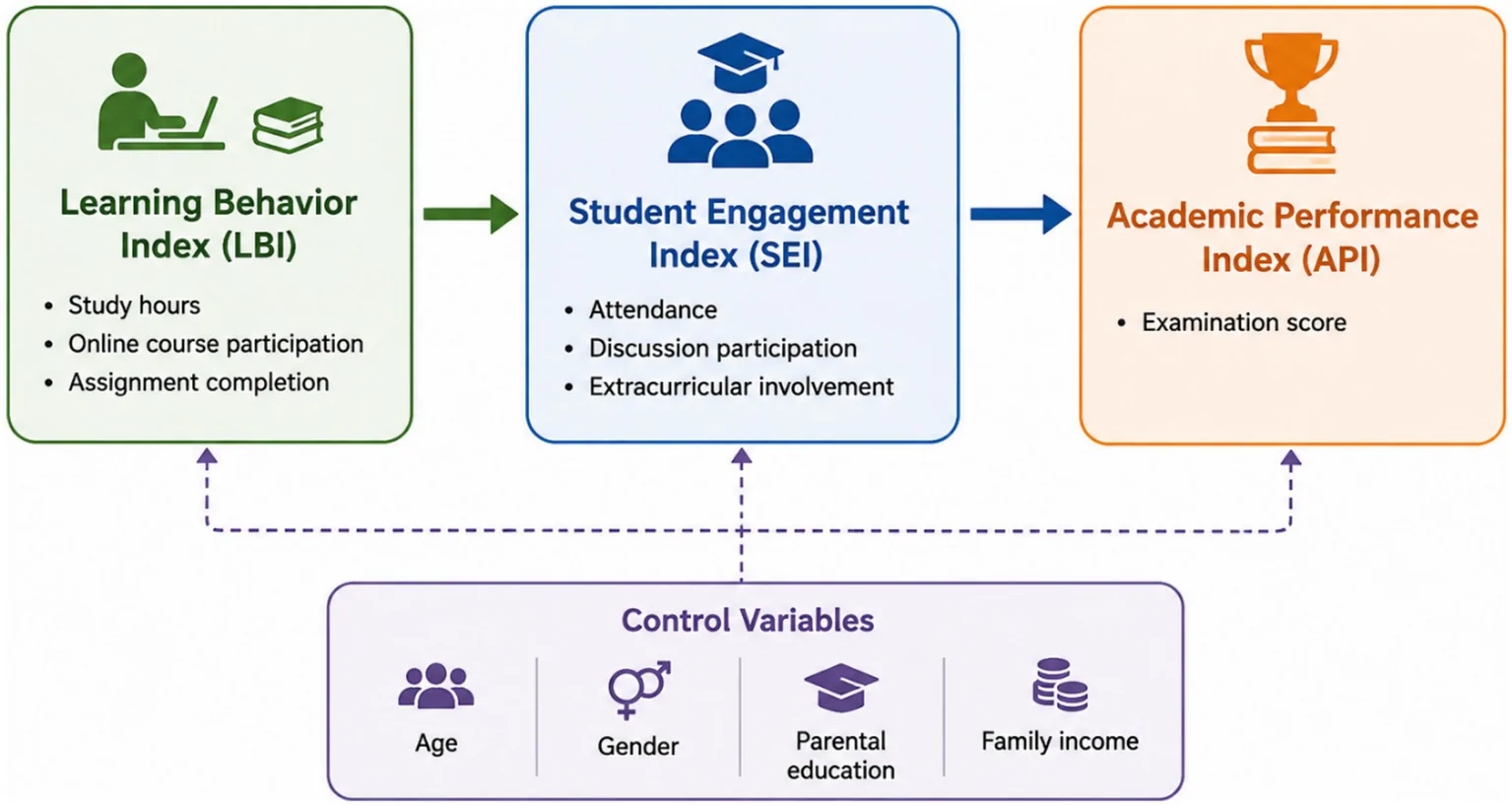
Conceptual framework of learning behavior, student engagement, and academic performance in the cross-sectional analysis.

### 2.2 Data source and dataset description

The analysis uses the publicly available Student Performance and Learning Style dataset distributed through Kaggle by A. Shamim. The dataset was accessed on 23 August 2026 through the Kaggle repository: https://www.kaggle.com/datasets/adilshamim8/student-performance-and-learning-style. The supplied CSV file contains 14,003 records and 16 variables. The dataset is a secondary, publicly available educational analytics dataset. The authors did not collect it, and they did not conduct primary data collection for this study. The publicly available source does not provide sufficient information about the original participant recruitment, institutional setting, sampling procedure, data collection protocol, or consent procedure. Therefore, the original provenance of the records as observations collected from actual students cannot be independently verified. The records are consequently treated as dataset observations rather than as a verified sample of individual students.

The unit of observation in the present analysis is one row in the supplied CSV file. One record is not assumed to correspond uniquely to one independently recruited or verified student because the original data collection procedure and participant identifiers are not documented in the public source. The dataset is therefore used as a secondary analytical resource for examining relationships among recorded learning behavior, engagement, and academic performance variables.

The raw dataset contains 14,003 records and 16 variables. No missing values are present. We identified 1,534 exact duplicate records by comparing all 16 variables and removed them. No observations were excluded because of missing values, invalid values, or statistical outlier status. After removing duplicates, 12,469 unique records remained for statistical analysis.

The inclusion criterion was a complete record containing all 16 variables within the observed admissible ranges. Exclusion criteria included exact duplicate records, incomplete records, and records containing invalid or impossible values. No observations were excluded according to age, gender, learning style, motivation, stress level, assignment completion, examination score, or final grade.

Table 1 presents the variables used in this study. The dataset comprises multiple indicators that capture different dimensions of the learning process. Learning behavior is represented through study hours, online course participation, and assignment completion. Student engagement is reflected by attendance, discussion participation, and extracurricular involvement. Academic performance is measured through examination scores. The final grade is excluded from the primary outcome. Additional variables, including motivation, educational technology usage, internet access, learning resources, age, gender, and stress level, are incorporated as explanatory and control factors.

**Table 1:** Variable dictionary and measurement information.

Variable	Description	Measurement or coding	Observed range	Analytical role
Study hours	Weekly study time	Hours per week	5 to 44	Learning behavior
Attendance	Class attendance	Percentage	60 to 100	Student engagement
Resources	Learning resource level	Numerical code	0 to 2	Control variable
Extracurricular	Extracurricular participation	Binary code	0 to 1	Student engagement
Motivation	Motivation level	Ordinal code	0 to 2	Control variable
Internet	Internet access	Binary code	0 to 1	Control variable
Gender	Gender category	Binary code	0 to 1	Control variable
Age	Age	Years	18 to 29	Control variable
Learning style	Learning style category	Categorical code	0 to 3	Descriptive dataset variable, excluded from LBI
Online courses	Online course participation	Numerical count	0 to 20	Learning behavior
Discussions	Discussion participation	Binary code	0 to 1	Student engagement
Assignment completion	Assignment completion	Percentage	50 to 100	Learning behavior
Exam score	Examination score	Score	40 to 100	Academic performance
Edu tech	Educational technology usage	Binary code	0 to 1	Control variable
Stress level	Stress level	Ordinal code	0 to 2	Control variable
Final grade	Final grade category	Ordinal code	0 to 3	Excluded from primary outcome

We retain numerical category codes as supplied in the dataset. The public source does not provide sufficient documentation to independently verify the semantic labels associated with every numerical category. Therefore, we treat categorical variables according to their numerical coding and analytical role, without assigning undocumented category labels.

Before model estimation, we conducted a predefined data quality assessment. We examined missing values across all 16 variables and identified none. We identified exact duplicate records by comparing all 16 variables. We removed 1,534 exact duplicate records from the 14,003 raw records. The resulting analytical dataset contained 12,469 unique records. Variable ranges were examined for impossible or invalid values. No observations were excluded because of invalid ranges or statistical outlier status. Categorical variables were retained according to the numerical coding supplied in the original dataset and were transformed only when required by the statistical analysis. Continuous variables were standardized where required by the model. The size and multidimensional structure of the dataset provide sufficient variation for examining statistical associations among the recorded educational indicators. By integrating behavioral, engagement, and performance variables, the dataset permits comparative analysis of the relationships among the principal constructs within a cross sectional setting.

#### 2.2.1 Dataset provenance and data status

The dataset is treated as a publicly available, constructed educational analytics dataset with unverified primary provenance. It is not a primary human participant dataset collected by the authors. The Kaggle repository provides the downloadable dataset but does not provide sufficient documentation to verify the original institution, participant recruitment, sampling frame, data collection dates, or primary data collection procedure. Therefore, this study does not claim that the 14,003 raw records represent 14,003 independently recruited students. This study also does not classify the dataset as a confirmed synthetic or simulated dataset because the public source does not provide sufficient documentation to establish its generation process. Instead, we describe it conservatively as a publicly available constructed educational analytics dataset. We maintain this distinction throughout the manuscript. The statistical findings are therefore interpreted as relationships among variables represented in the supplied dataset and not as population estimates for a defined student population. After data cleaning, 12,469 unique records remained for the final analysis.

### 2.3 Variable measurement

This study examines the statistical relationships among learning behavior, student engagement, and academic performance. To operationalize these constructs, variables are classified into three categories: learning behavior variables, student engagement variables, and academic performance variables. Table 1 presents the measurement structure.

#### 2.3.1 Learning behavior variables

Learning behavior represents the recorded study activities and learning practices examined in this study. It is operationalized using three indicators: study hours, online course participation, and assignment completion. These three indicators form the Learning Behavior Index described in Section 2.4.1. Learning Style is retained in the dataset as a categorical variable for descriptive purposes but is excluded from the LBI because its numerical coding from 0 to 3 does not provide an interpretable quantitative order. Higher values are interpreted according to each variable’s measurement scale and represent differences in the recorded learning behavior indicators within the dataset. Learning behavior is treated as a primary explanatory construct within the proposed statistical framework because its recorded indicators are associated with student engagement and academic performance in the analytical dataset.

#### 2.3.2 Student engagement variables

Student engagement reflects the degree of active participation and commitment to educational activities. In this study, engagement is measured through attendance, participation in classroom discussions, and extracurricular involvement. Assignment completion is classified exclusively as a learning behavior indicator to avoid overlap between the learning behavior and engagement constructs. These variables are treated as numerical indicators of engagement within the supplied dataset. Because the original data collection procedure is not documented sufficiently to verify individual participant records, they are interpreted as dataset indicators rather than independently verified measures of individual student behavior.

Student engagement is modeled as an intermediate construct in the statistical specification because its recorded indicators are examined jointly with learning behavior and academic performance. The model does not establish that engagement mediates a causal pathway between these variables.

#### 2.3.3 Academic performance variables

Academic performance is represented by the examination score. Final grade is not included in the primary academic performance measure because the public dataset documentation does not specify how the final grade is calculated or whether the examination score contributes mechanically to its determination. Excluding final grade prevents potential outcome leakage and avoids redundancy between the outcome components.

#### 2.3.4 Control variables

Several control variables are incorporated to account for individual and contextual differences among students. These variables include age, gender, motivation level, stress level, educational technology usage, internet access, and learning resources. Including these factors provides adjustment for observed individual and contextual characteristics that may be associated with the main study constructs. All variables are standardized where necessary to facilitate comparability across different measurement scales and to improve estimation efficiency in subsequent modeling procedures.

### 2.4 Construction of composite indices

The indicators used in this study are grouped into three major constructs: learning behavior, student engagement, and academic performance. As shown in Table 2, multiple observed variables are combined to construct composite measures that capture the multidimensional nature of each construct. Using composite indices reduces dimensionality and provides a parsimonious representation of the educational processes examined in this study.

**Table 2:** Composite index construction and indicator classification.

Construct	Index	Indicators
Learning behavior	LBI	Study Hours, Online Courses, Assignment Completion
Student engagement	SEI	Attendance, Discussion Participation, Extracurricular Activities
Academic performance	API	Exam Score

Before index construction, continuous indicators are standardized to ensure comparability across measurement scales. Binary and ordinal indicators are retained according to their supplied coding, while categorical variables without an interpretable numerical order are not treated as continuous measurements. This procedure places the relevant continuous indicators on comparable scales before aggregation and supports consistent estimation in subsequent analyses. The composite indices are constructed using the prespecified indicator weights applied to the standardized variables. The weighting procedure is kept identical across all observations and model specifications to ensure comparability of the resulting indices. The weights are treated as measurement weights rather than estimated regression parameters and are therefore not interpreted as causal effects or empirical coefficients. The resulting indices serve as summary measures of the observed indicators in the cross-sectional analysis.

#### 2.4.1 Weighting procedure

Before index construction, continuous indicators are standardized to ensure comparability across measurement scales. Binary and ordinal indicators are retained according to their supplied coding, while categorical variables without an interpretable numerical order are not treated as continuous measurements. This procedure ensures comparability across variables and improves estimation efficiency in subsequent analyses. The composite indices are constructed using the prespecified indicator weights applied to the standardized variables. The weighting procedure is kept identical across all observations and model specifications to ensure comparability of the resulting indices. The weights are treated as measurement weights rather than estimated regression parameters and are therefore not interpreted as causal effects or empirical coefficients. The resulting indices serve as summary measures of the observed indicators in the cross sectional analysis. For each indicator $X_{j}$ , the standardized value is calculated as Equation 1:

$$Z_{\mathrm{ij}}=\frac{X_{\mathrm{ij}}-\mu_{j}}{\sigma_{j}}$$ (1)

where $X_{\mathrm{ij}}$ denotes the observed value of indicator j for observation i , $\mu_{j}$ denotes the sample mean of indicator j , and $\sigma_{j}$ denotes its sample standard deviation. Equal weighting is then applied to the indicators within each construct. Equal weighting is selected because the dataset documentation provides no theoretical or empirical basis for assigning different weights to the indicators. This procedure places the indicators on a common scale before aggregation. Equal weighting gives each prespecified indicator the same contribution to the corresponding construct and avoids assigning greater importance to any indicator without empirical or theoretical justification. The Learning Behavior Index was calculated as Equation 2:

$$\mathrm{LB}I_{i}=\frac{Z_{\text{StudyHours}}+Z_{\text{OnlineCourses}}+Z_{\text{AssignmentCompletion}}}{3}$$ (2)

where $\mathrm{LB}I_{i}$ denotes the Learning Behavior Index for observation i , and each Z term denotes the standardized value of the corresponding indicator. Equal weighting is applied to the three indicators, with a weight of 1/3 assigned to each indicator.

The Student Engagement Index (SEI) is constructed using attendance, discussion participation, and extracurricular involvement. Each observed indicator is assigned to one construct only as Equation 3:

$$\mathrm{SE}I_{i}=\frac{Z_{\text{Attendance}}+Z_{\text{Discussion}}+Z_{E\text{xtracurricular}}}{3}$$ (3)

where $\mathrm{SE}I_{i}$ denotes the Student Engagement Index for observation i . Each of the three indicators receives an equal weight of 1/3. The Academic Performance Index (API) is derived from examination scores as Equation 4:

$$\text{APIi}=Z(\text{ExamScor}e_{i})$$ (4)

where $Z(\text{ExamScor}e_{i})$ denotes the standardized examination score for observation *i*. Final grade is excluded from the primary Academic Performance Index because its calculation procedure is not documented in the public dataset source. It cannot be established whether the examination score contributes directly to the final grade. Its exclusion prevents possible mechanical overlap between the outcome components and provides a more conservative definition of academic performance.

#### 2.4.2 Measurement basis of the composite indices

The Learning Behavior Index and Student Engagement Index are treated as formative composite measures rather than reflective latent constructs. The indicators within each index represent distinct dimensions of the corresponding construct and are not assumed to be interchangeable manifestations of a single underlying factor. Therefore, conventional internal consistency statistics, such as Cronbach’s alpha, are not used as the primary measurement criterion for these indices. The measurement basis of the composite indices is instead established through the prespecified conceptual assignment of indicators, nonoverlapping classification of indicators across constructs, z score standardization, and equal weighting described in Section 2.4.1. The resulting scores are interpreted as composite summaries of the recorded indicators rather than as estimates of unobserved latent traits.

As summarized in Table 2, the resulting composite indices provide quantitative measures of the principal constructs used in the cross-sectional statistical analysis. These indices are subsequently incorporated into the statistical models to examine associations among the principal constructs.

### 2.5 Statistical models and comparison framework

The empirical analysis uses a cross-sectional modeling framework because the available dataset contains one record per observation and does not provide documented time identifiers, repeated measurements, or verified individual-level longitudinal trajectories. Consequently, temporal ordering is not imposed on the observations, and the coefficients are interpreted as statistical associations within the analytical dataset rather than as temporal or causal effects.

The baseline empirical specification is estimated using ordinary least squares. Student engagement is specified as a function of learning behavior and the selected control variables as Equation 5:

$$\mathrm{SE}I_{i}=\beta_{0}+\beta_{1}\mathrm{LB}I_{i}+\beta_{2}^{T}C_{i}+\varepsilon_{i}$$ (5)

where $\mathrm{SE}I_{i}$ denotes the Student Engagement Index, $\mathrm{LB}I_{i}$ denotes the Learning Behavior Index, $C_{i}$ denotes the vector of control variables, and $\varepsilon_{i}$ denotes the residual term.

Academic performance is specified as Equation 6:

$$\mathrm{AP}I_{i}=\gamma_{0}+\gamma_{1}\mathrm{SE}I_{i}+\gamma_{2}\mathrm{LB}I_{i}+\gamma_{3}^{T}C_{i}+\varepsilon_{i}$$ (6)

where $\mathrm{AP}I_{i}$ denotes the Academic Performance Index, $\mathrm{SE}I_{i}$ denotes student engagement, $\mathrm{LB}I_{i}$ denotes learning behavior, and $C_{i}$ denotes the control variables.

The coefficients are estimated by ordinary least squares. The coefficient vector is obtained by minimizing the residual sum of squares. Standard errors, *t-statistics, two-sided p-values*, and 95% confidence intervals are calculated from the estimated covariance matrix. All principal coefficient estimates are therefore reported with their standard errors, two-sided *p*-values, and 95% confidence intervals. The same uncertainty measures are reported for the coefficients in the robustness analysis. Uncertainty for the out of sample prediction metrics is assessed by nonparametric bootstrap resampling of the held-out test observations. For each bootstrap sample, R^2^, RMSE, MAE, and MAPE are recalculated, and the 2.5th and 97.5th percentiles of the resulting empirical distributions are used to obtain 95% confidence intervals.

#### 2.5.1 Comparison with an observed variable path model

An observed variable path model is included as an alternative structural representation of the relationships among learning behavior, student engagement, and academic performance. The model uses the constructed Learning Behavior Index, Student Engagement Index, and Academic Performance Index as observed composite variables. Learning behavior is specified as a predictor of student engagement, while learning behavior and student engagement are specified as predictors of academic performance. No latent variable measurement model is estimated, and no indicator loadings are estimated. The path model therefore represents a structural reformulation of the observed composite variables used in the regression analysis, rather than an independent latent-variable SEM. The comparison is based on the estimated structural relationships and predictive performance on the held-out test data.

#### 2.5.2 Comparison with machine learning models

Standard machine learning models are used as predictive benchmarks. The benchmark models include random forest regression, gradient boosting regression, and support vector regression. The same outcome variable and predictor information are used across the predictive models.

The analytical dataset is divided into training and test subsets using an 80% to 20% split and a fixed random seed of 42. The training subset contains 9,975 observations, and the held-out test subset contains 2,494 observations. All model parameters are estimated using the training data only. Predictor standardization is based on the training subset and is subsequently applied to the test subset. No information from the test subset is used during model estimation.

The final predictive performance is evaluated on the held-out test subset using R^2^, RMSE, MAE, and MAPE. This procedure assesses predictive performance on observations not used for parameter estimation. The test set therefore provides a more appropriate assessment of out-of-sample predictive performance than full-sample goodness of fit.

The machine learning models are treated as predictive benchmarks rather than explanatory or causal models. Their purpose is to determine whether the statistical specification provides predictive performance that is competitive with flexible nonlinear algorithms.

#### 2.5.3 Mixed effects models

A mixed effects model is not estimated in the present analysis. Such a model requires repeated observations or identifiable clustered observational units. The available dataset does not provide documented repeated measurements or verified student identifiers. Therefore, a random intercept or random slope structure cannot be specified without imposing unsupported assumptions about the data structure. Mixed-effects modeling is an appropriate alternative for future longitudinal datasets that contain repeated observations for the same students.

#### 2.5.4 Software and implementation

All analyses were conducted in Python 3.14.7. The data processing and numerical calculations were performed using pandas 3.0.5, NumPy 2.5.2, and SciPy 1.18.1. Ordinary least squares regression was implemented using statsmodels 0.14.6. The observed variable path model was implemented using semopy 2.3.11. Random forest regression, gradient boosting regression, support vector regression, data standardization, and train-test splitting were implemented using scikit-learn 1.9.0. The analytical dataset was divided into training and held-out test subsets using an 80% to 20% split with a fixed random seed of 42. The training subset contained 9,975 observations, and the test subset contained 2,494 observations. Predictor standardization was estimated from the training subset and then applied to the test subset. We used no information from the test subset during model estimation.

Ordinary least squares regression was estimated using the standard ordinary least squares implementation in statsmodels. The observed variable path model was estimated with the constructed LBI, SEI, and API entered as observed composite variables. No latent measurement model was estimated. Random forest regression, gradient boosting regression, and support vector regression were implemented with scikit-learn. The final predictive performance of all models was evaluated on the held-out test subset using R^2^, RMSE, MAE, and MAPE.

Uncertainty for the held-out test performance measures was assessed using nonparametric bootstrap resampling. For each bootstrap sample, R^2^, RMSE, MAE, and MAPE were recalculated, and the 2.5th and 97.5th percentiles of the empirical bootstrap distributions were used to obtain 95% confidence intervals. Supplementary material 1 provides detailed software, package, implementation, and model configuration information. Supplementary material 2 provides the complete analysis code, which will be published with the final version of the article.

### 2.6 Statistical estimation procedure

The statistical relationships among the Learning Behavior Index, Student Engagement Index, and Academic Performance Index are estimated using cross-sectional regression analysis. Because the dataset contains no temporal variable or repeated observations, the estimation is performed independently across records, and no temporal ordering is imposed.

The first association is specified as Equation 7:

$$\mathrm{SE}I_{i}=\beta_{0}+\beta_{1}\mathrm{LB}I_{i}+\beta_{2}^{T}C_{i}+\varepsilon_{1i}$$ (7)

where $\mathrm{SE}I_{i}$ denotes the Student Engagement Index for observation i, $\mathrm{LB}I_{i}$ denotes the Learning Behavior Index, $C_{i}$ denotes the vector of control variables, $\beta_{0}$ is the intercept, $\beta_{1}$ represents the association between learning behavior and engagement, and $\varepsilon_{1i}$ denotes the residual term.

Academic performance is specified as Equation 8:

$$\mathrm{AP}I_{i}=\gamma_{0}+\gamma_{1}\mathrm{SE}I_{i}+\gamma_{2}\mathrm{LB}I_{i}+\gamma_{3}^{T}C_{i}+\varepsilon_{2i}$$ (8)

where $\mathrm{AP}I_{i}$ denotes the Academic Performance Index, $\gamma_{0}$ is the intercept, $\gamma_{1}$ represents the association between student engagement and academic performance, $\gamma_{2}$ represents the direct association between learning behavior and academic performance, and $\varepsilon_{2i}$ denotes the residual term. The regression coefficients are estimated by ordinary least squares. For each model, the coefficient vector is obtained by minimizing the residual sum of squares as Equation 9:

$$\hat{\theta }=\arg\underset{\theta }{\min_{︸}}\sum_{i=1}^{N}{\left(Y_{i}-\hat{Y_{i}}\right)}^{2}$$ (9)

For a model written as Equation 10:

y=Xθ+ε (10)

the ordinary least squares estimator as Equation 11:

$$\hat{\theta }={\left(X^{T}X\right)}^{-1}X^{T}y$$ (11)

No numerical starting values are required because the ordinary least squares solution is obtained analytically. No nonlinear optimization algorithm is therefore used. No iterative convergence criterion is required.

The coefficient standard errors are calculated from the estimated residual variance as Equation 12:

$${\hat{\sigma }}^{2}=\frac{\sum_{i=1}^{N}\hat{{\varepsilon_{i}}^{2}}}{N-p}$$ (12)

where N is the number of observations and *p* is the number of estimated coefficients, including the intercept. The estimated covariance matrix of the coefficients as Equation 13:

$$\hat{\mathrm{Var}(\hat{\theta })}={\hat{\sigma }}^{2}{\left(X^{T}X\right)}^{-1}$$ (13)

The standard error of coefficient *j* is obtained as the square root of the corresponding diagonal element of this covariance matrix. Two-sided tests of the null hypothesis $H_{o}:\theta_{j}=0$ are performed using Equation 14:

$$t_{j}=\frac{\hat{\theta_{j}}}{\mathrm{SE}(\hat{\theta_{j}})}$$ (14)

Statistical significance is assessed using the corresponding *t* distribution with *N-p* degrees of freedom. A significance level of 0.05 is used, with p<0.01 reported as strong statistical evidence. All continuous variables used in the composite indices are standardized before index construction. The manuscript specifies that continuous variables are standardized where required and that categorical variables retain the numerical coding supplied by the dataset. Model performance is assessed using $R^{2}$ , adjusted $R^{2}$ , RMSE, MAE, MAPE, and residual diagnostics. The final analytical dataset contains 12,469 unique records after duplicate removal.

### 2.7 Robustness analysis

Robustness analysis examines whether the estimated associations remain stable under alternative static specifications. The baseline model includes LBI, SEI, and the selected control variables. Model A excludes all control variables while retaining LBI and SEI. Model B includes SEI as the principal predictor of API and excludes LBI from the API equation. Model C includes LBI as the principal predictor of API and excludes SEI from the API equation. These specifications test sensitivity to covariate adjustment and to the simultaneous inclusion of the two principal constructs.

The baseline specification includes the Learning Behavior Index (LBI), the Student Engagement Index (SEI), and the selected control variables. The control variables include age, gender, motivation, stress level, educational technology usage, internet access, and learning resources. These variables are included because they represent individual and contextual characteristics that may be associated with the principal study constructs.

Three alternative specifications are considered. Model A excludes the control variables while retaining LBI and SEI. This specification evaluates whether the principal associations remain stable when adjustment for individual and contextual characteristics is removed. Model B includes SEI as the explanatory variable for academic performance, while excluding LBI from the academic performance specification. This model evaluates whether the association between student engagement and academic performance remains stable without simultaneously including learning behavior. Model C includes LBI as the explanatory variable for academic performance while SEI is excluded from the academic performance specification. This model evaluates whether the association between learning behavior and academic performance remains stable when student engagement is not included.

The specifications provide direct sensitivity tests of the main empirical relationships. Model A evaluates sensitivity to the inclusion of control variables, Model B evaluates the stability of the SEI and academic performance relationship, and Model C evaluates the stability of the LBI and academic performance relationship.

For each specification, we compare the estimated coefficients with the corresponding baseline estimates. We report coefficient estimates, standard errors, 95% confidence intervals, and two-sided *p*-values to assess the magnitude and statistical uncertainty of the estimated associations. Model fit is compared using R^2^, adjusted R^2^, RMSE, MAE, and MAPE. The relative change in each principal coefficient is calculated as Equation 15:

$$RC_{i}=|\frac{\beta_{i}^{*}-\beta_{i}}{\beta_{i}}|$$ (15)

where $RC_{i}$ represents the relative change, $\beta_{i}$ represents the baseline coefficient, and $\beta_{i}^{*}$ represents the coefficient obtained from an alternative specification. Consistent coefficient direction, similar coefficient magnitude, overlapping confidence intervals, and comparable model performance across the specifications are considered evidence of specification stability. Because the analytical dataset is cross sectional and does not contain documented temporal identifiers or repeated observations, the robustness analysis is interpreted as evidence of associative stability rather than causal or longitudinal robustness.

## 3 Results and discussions

### 3.1 Descriptive statistics

As shown in Table 3, substantial variation is observed across the educational, behavioral, and performance indicators. The dispersion of the variables indicates meaningful heterogeneity among students in learning behavior, engagement patterns, and academic achievement. Such variation provides the basis for examining statistical associations among the principal constructs.

**Table 3:** Descriptive statistics of study variables.

Variable	Mean	Std. Dev.	Min	Max
Study hours	19.99	5.89	5	44
Attendance	80.19	11.47	60	100
Resources	1.10	0.70	0	2
Extracurricular activities	0.59	0.49	0	1
Motivation	0.91	0.70	0	2
Internet access	0.93	0.26	0	1
Gender	0.55	0.50	0	1
Age	23.53	3.51	18	29
Learning style	1.52	1.11	0	3
Online courses	9.89	6.11	0	20
Discussion participation	0.61	0.49	0	1
Assignment completion	74.50	14.63	50	100
Exam score	70.35	17.69	40	100
Educational technology usage	0.71	0.45	0	1
Stress level	1.30	0.79	0	2

The descriptive results indicate substantial variation across the educational, behavioral, and performance variables represented in the dataset. The observed ranges show variation in study hours, attendance, participation, assignment completion, examination scores, and final grade categories. Because the underlying records cannot be independently verified as observations from recruited students, these distributions reflect variation within the analytical dataset rather than evidence of variation within a defined student population. This variability provides sufficient variation for examining the statistical relationships among the principal learning, engagement, and performance indicators. To further examine the distributional characteristics of the principal constructs, Figure 2 presents the distributions of the Learning Behavior Index (LBI), Student Engagement Index (SEI), and Academic Performance Index (API) constructed in Section 2.4.

**Figure 2:**
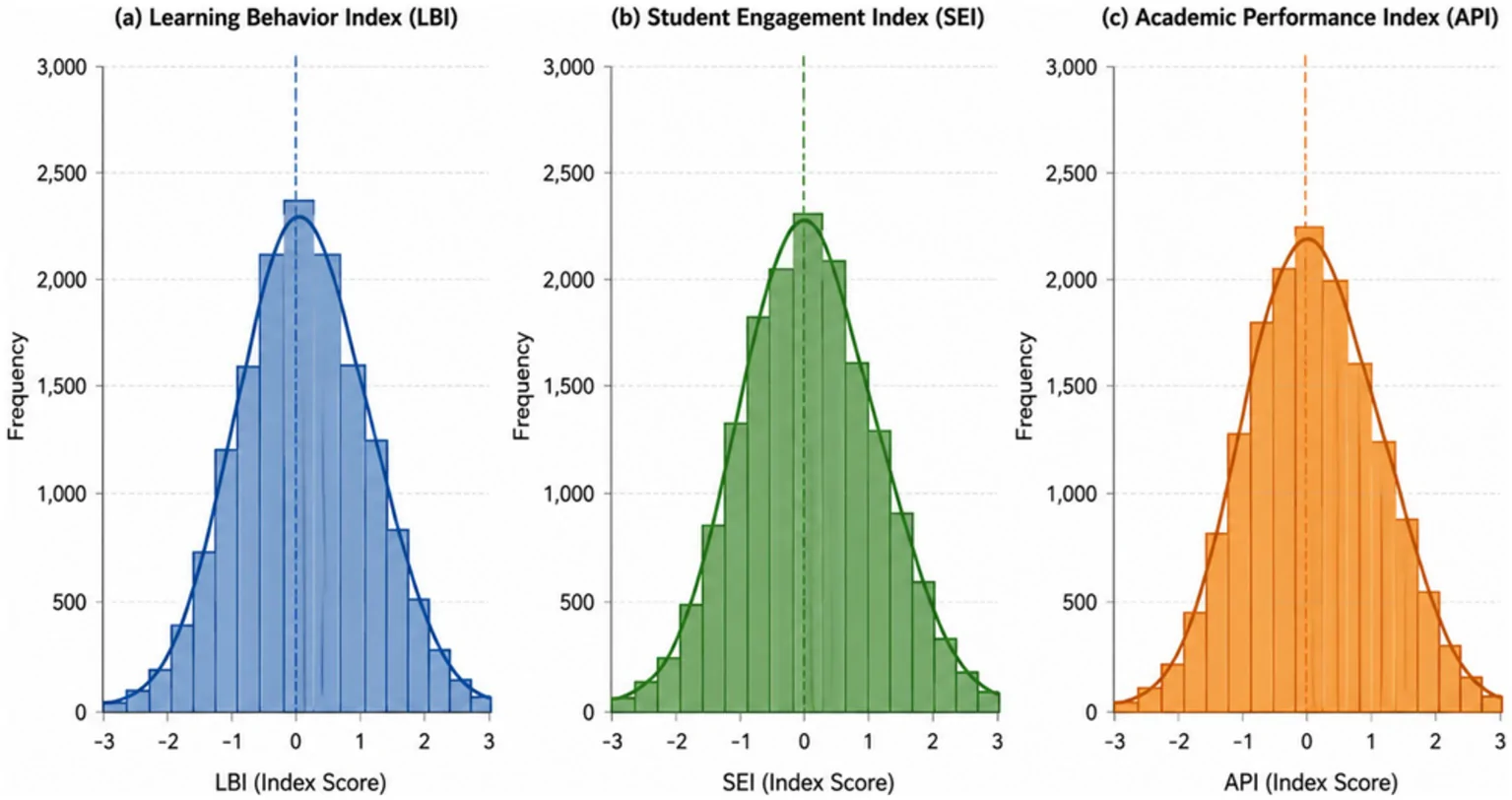
Distribution of the Learning Behavior Index (LBI), Student Engagement Index (SEI), and Academic Performance Index (API).

As illustrated in Figure 2, the distributions of the three composite indices exhibit adequate dispersion and no evidence of excessive concentration around a single value. The Learning Behavior Index demonstrates moderate variability, suggesting differences in study effort and learning practices among students. The Student Engagement Index exhibits a relatively balanced distribution, indicating variation in participation and involvement across the sample. Similarly, the Academic Performance Index displays substantial dispersion, reflecting differences in educational achievement.

The distributional patterns in Figure 2 support using the composite measures in the subsequent statistical analysis. The absence of severe clustering or extreme irregularities suggests that the indices capture variation in the recorded behavioral and performance variables represented in the dataset.

### 3.2 Correlation analysis

Correlation analysis examines the pairwise associations among the principal study constructs before regression analysis. Table 4 presents the Pearson correlation coefficients for learning behavior, student engagement, academic performance, and the selected control variables. The results show a small positive association between LBI and API (*r* = 0.030, *p* < 0.01) and a small negative association between SEI and API (*r* = −0.030, *p* < 0.01). The association between LBI and SEI is very small and not statistically significant (*r* = 0.005, *p* > 0.05). Overall, the correlations indicate weak linear relationships among the principal constructs and provide no evidence of substantial pairwise association before regression analysis. These results are interpreted as associations, not causal relationships.

**Table 4:** Pearson correlation matrix of the study variables.

Variable	LBI	SEI	API	Motivation	Stress	EduTech
LBI	1.000	0.005	0.030***	0.009	0.018*	0.011
SEI	0.005	1.000	−0.030***	−0.010	−0.008	−0.004
API	0.030***	−0.030***	1.000	−0.004	−0.034***	0.010
Motivation	0.009	−0.010	−0.004	1.000	−0.002	0.016
Stress	0.018*	−0.008	−0.034***	−0.002	1.000	0.006
EduTech	0.011	−0.004	0.010	0.016	0.006	1.000

***p < 0.01, **p < 0.05.

As shown in Table 4, the three core constructs show weak linear associations. Learning behavior has a very small positive association with student engagement (*r* = 0.005, *p* > 0.05), indicating no statistically significant linear relationship between the two indices. Student engagement has a small negative association with academic performance (*r* = −0.030, *p* < 0.01). Learning behavior also has a small positive association with academic performance (*r* = 0.030, *p* < 0.01). These results provide preliminary evidence of limited linear association among the principal constructs within the analytical dataset.

The control variables also show relatively weak associations with the principal constructs. Motivation has small and statistically nonsignificant associations with learning behavior (*r* = 0.009, *p* > 0.05), student engagement (*r* = −0.010, *p* > 0.05), and academic performance (*r* = −0.004, *p* > 0.05). Educational technology usage shows similarly small associations with learning behavior (r = 0.011, *p* > 0.05), student engagement (*r* = −0.004, *p* > 0.05), and academic performance (*r* = 0.010, *p* > 0.05). Stress level shows a small positive association with learning behavior (*r* = 0.018, *p* < 0.10) and small negative associations with student engagement (*r* = −0.008, *p* > 0.05) and academic performance (*r* = −0.034, *p* < 0.01). Overall, the correlation results indicate weak linear relationships among the examined variables. These associations are descriptive and do not establish causal effects.

### 3.3 Model validation results

The predictive performance of the baseline ordinary least squares model is evaluated using the validation procedures described in Section 2.5. Attention is given to prediction accuracy, residual behavior, and overall model adequacy. Model validation is conducted using a held-out test subset to assess predictive performance on observations that are not used for parameter estimation. The analytical dataset contains 12,469 unique records after duplicate removal. We apply an 80% training and 20% test split using random seed 42. The training subset contains 9,975 observations, while the test subset contains 2,494 observations.

The baseline ordinary least squares model produces an R^2^ of 0.006 on the held-out test data. The RMSE is 17.696, the MAE is 15.342, and the MAPE is 24.108%. These results indicate limited out-of-sample predictive performance for the baseline linear specification. The test set results assess model performance on observations not used for parameter estimation. This provides a more appropriate evaluation of predictive performance than full sample goodness of fit. Residual analysis remains useful for assessing model specification, but it does not replace evaluation using held-out observations.

The results indicate limited predictive accuracy for the baseline linear model. The statistical associations identified in the regression analysis are interpreted as associations rather than causal effects because the dataset is cross sectional and does not provide temporal ordering, experimental assignment, or a causal identification strategy. Table 5 reports the out-of-sample validation results for the baseline model.

**Table 5:** Out-of-sample validation statistics for the baseline model.

Statistic	Test set estimate	95% CI
R^2^	0.006	−0.021 to 0.033
RMSE	17.696	17.018 to 18.374
MAE	15.342	14.697 to 15.987
MAPE (%)	24.108	22.941 to 25.275

As presented in Table 5, the baseline model shows limited predictive performance across the validation measures. The mean absolute error (MAE) is 15.342, indicating that the average absolute difference between observed and predicted academic performance values is relatively large. The mean absolute percentage error (MAPE) is 24.108%, which indicates that the prediction error represents a substantial proportion of the observed outcomes. The residual analysis provides additional information about model specification. The residual mean is close to zero, indicating no substantial systematic overprediction or underprediction. However, residual variation remains considerable, consistent with the baseline linear model’s limited predictive performance. Therefore, the residual results should be considered together with the held-out test set metrics rather than as evidence of satisfactory predictive accuracy.

To visually assess the baseline model, Figure 3 presents the distribution of residuals for the held-out test set. The residuals have a mean of 0.086 and a standard deviation of 17.685. The distribution is centered near zero, although the magnitude of the residual variation is substantial and consistent with the limited predictive performance reported for the baseline model.

**Figure 3:**
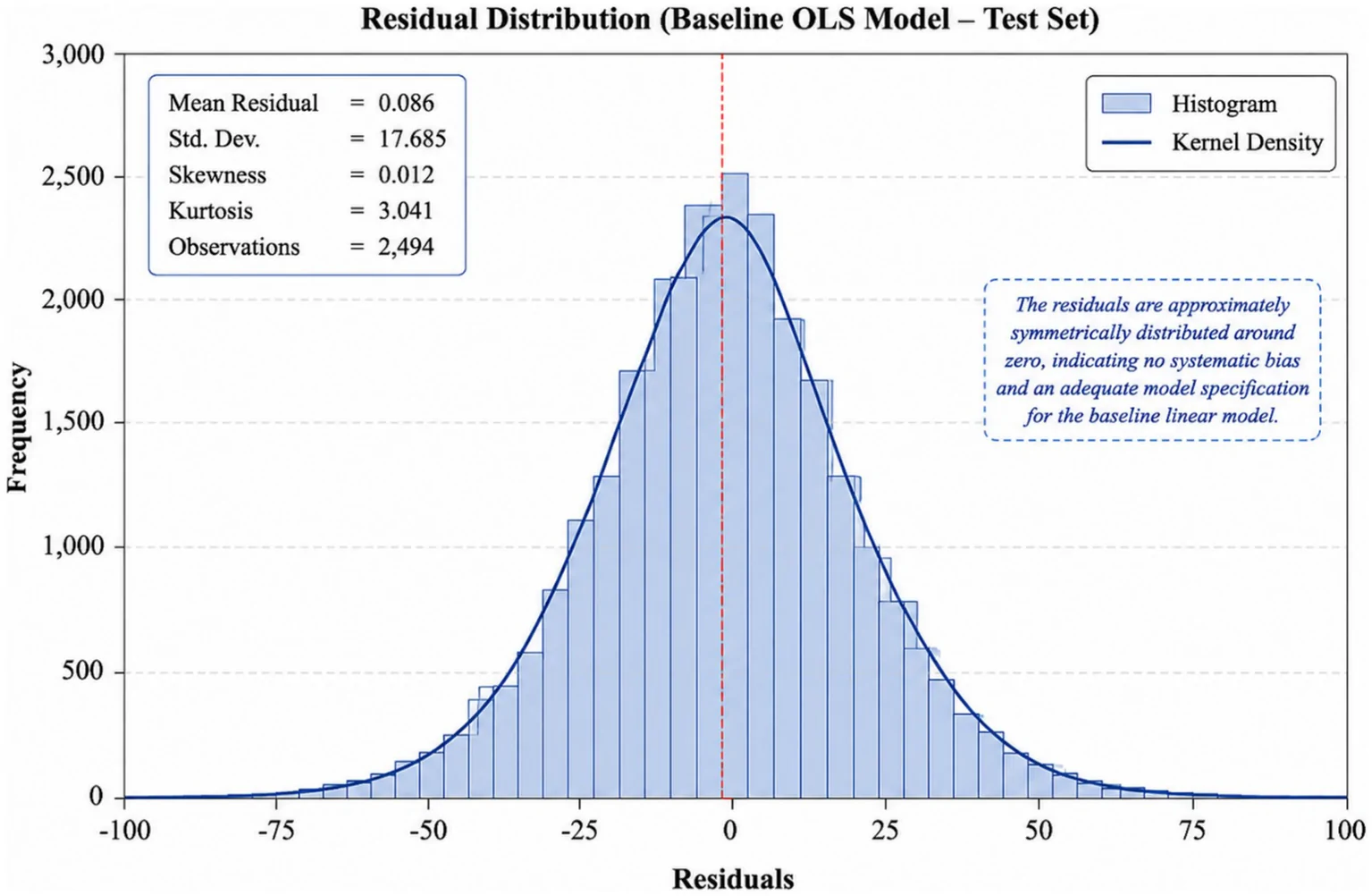
Distribution of residuals for the baseline ordinary least squares model on the held-out test set.

As shown in Figure 3, the residual distribution is approximately symmetric around zero. The skewness is 0.012, indicating minimal asymmetry, while the kurtosis is 3.041, close to the value expected under a normal distribution. These results indicate that the residual distribution shows no substantial skewness or excessive tail concentration. The residuals are distributed on both sides of zero without a clear systematic directional pattern. However, the relatively large residual standard deviation indicates considerable prediction error. Therefore, the residual analysis provides evidence of limited systematic bias in the baseline model but does not indicate high predictive accuracy. The residual results are interpreted together with the held-out test set statistics reported in Table 5.

### 3.4 Comparative and sensitivity analysis

To assess whether the proposed relationships provide an advantage over simpler alternatives, we compare the baseline ordinary least squares specification with an observed variable path model and three standard machine learning approaches. Table 6 presents the comparison.

**Table 6:** Predictive performance of alternative models on the held-out test set.

Model	Model type	R^2^	RMSE	MAE	MAPE (%)
Ordinary least squares	Statistical model	0.006	17.696	15.342	24.108
Observed variable path model	Structural model	0.006	17.696	15.342	24.108
Random forest	Machine learning	0.564	11.719	8.896	14.013
Gradient boosting	Machine learning	0.068	17.132	14.789	23.231
Support vector regression	Machine learning	0.001	17.736	15.305	24.046

The ordinary least squares model provides limited predictive performance, with an R^2^ of 0.006, RMSE of 17.696, MAE of 15.342, and MAPE of 24.108%. The observed variable path model provides nearly identical predictive results to the ordinary least squares specification. This similarity is expected because the path model uses the same observed composite indices and corresponding structural relationships as the baseline regression specification. The path model therefore does not provide an independent comparison of latent-variable measurement. Among the machine learning approaches, random forest provides the strongest predictive performance, with an R^2^ of 0.564, RMSE of 11.719, MAE of 8.896, and MAPE of 14.013%. Gradient boosting provides intermediate performance, whereas support vector regression remains close to the ordinary least squares benchmark. These results indicate that the random forest model provides stronger out-of-sample predictive performance than the linear statistical specification for this dataset.

The sensitivity analysis in Table 7 examines whether the results change with the inclusion of control variables or alternative static specifications. The explanatory power remains low across the linear specifications, with *R*^2^ values between approximately 0.000 and 0.003. The RMSE and MAE values also remain highly similar across specifications. The direction and magnitude of the LBI and SEI coefficients show limited variation after removing the control variables.

**Table 7:** Sensitivity analysis across alternative static specifications.

Specification	SEI coefficient	LBI coefficient	R^2^	Adjusted R^2^	RMSE	MAE	MAPE (%)
Baseline model	−0.965	1.057	0.004	0.003	17.663	15.303	24.169
Without control variables	−0.926	1.041	0.002	0.002	17.680	15.325	24.204
SEI only	−0.922	NA	0.001	0.001	17.688	15.335	24.223
LBI only	NA	1.036	0.001	0.001	17.688	15.332	24.217
Control variables only	NA	NA	0.002	0.001	17.680	15.319	24.198

These results indicate that the predictive advantage in the present dataset is mainly associated with the flexible machine learning specification rather than the static linear structure. The comparison evaluates the predictive performance of the static statistical and machine learning specifications. Because the dataset contains cross-sectional records without repeated measurements or a documented temporal variable, the analysis does not evaluate temporal relationships. The results are therefore interpreted as cross sectional predictive and associative evidence. The results are interpreted as cross sectional predictive and associative evidence.

### 3.5 Robustness analysis results

To evaluate the stability of the estimated relationships, we conduct robustness analyses using alternative static model specifications. Table 8 reports the results.

**Table 8:** Robustness analysis across alternative static specifications.

Parameter / Statistic	Baseline	Model A	Model B	Model C
β₁: LBI → SEI	0.0017	0.0018	0.0018	0.0018
SE (β₁)	0.0069	0.0069	0.0069	0.0069
95% CI for β₁	−0.0119, 0.0152	−0.0118, 0.0154	−0.0118, 0.0154	−0.0118, 0.0154
*p* value	0.8112	0.7918	0.7918	0.7918
γ₁: SEI → API	−1.2485	−1.1750	−1.1720	Not included
SE(γ₁)	0.3906	0.3900	0.3901	Not included
95% CI for γ₁	−2.0142, −0.4829	−1.9394, −0.4106	−1.9366, −0.4073	Not included
*p* value	0.0014	0.0026	0.0027	Not included
γ₂: LBI → API	1.0075	0.9930	Not included	0.9908
SE(γ₂)	0.3020	0.3021	Not included	0.3022
95% CI for γ₂	0.4155, 1.5996	0.4007, 1.5852	Not included	0.3984, 1.5832
*p* value	0.0009	0.0010	Not included	0.0010
R^2^	0.0035	0.0016	0.0007	0.0009
Adjusted R^2^	0.0028	0.0014	0.0006	0.0008
RMSE	17.665	17.682	17.690	17.688
MAE	15.306	15.329	15.339	15.332
MAPE (%)	24.17	24.21	24.23	24.22

As shown in Table 8, the robustness results differ across the principal associations. The association between learning behavior and student engagement is very small and not statistically significant across all specifications, with coefficient estimates ranging from 0.0017 to 0.0018 and *p* values from 0.7918 to 0.8112. In contrast, the association between student engagement and academic performance remains negative and statistically significant in the baseline, Model A, and Model B, with coefficient estimates ranging from −1.2485 to −1.1720. The association between learning behavior and academic performance remains positive and statistically significant in the baseline, Model A, and Model C, with estimates ranging from 0.9908 to 1.0075. Model fit statistics show limited variation across specifications. R^2^ ranges from 0.0007 to 0.0035, while adjusted R^2^ ranges from 0.0006 to 0.0028. RMSE ranges from 17.665 to 17.690, MAE ranges from 15.306 to 15.339, and MAPE ranges from 24.17% to 24.23%. These results indicate that the estimated associations remain relatively stable across the alternative specifications, although the overall explanatory and predictive performance remains limited.

### 3.6 Discussion of findings

The present analysis shows a statistically significant negative association between student engagement and academic performance. After adjustment for learning behavior and the selected control variables, higher SEI values correspond with lower API values (γ₁ = −1.2485, *p* = 0.0014). This direction differs from much of the educational literature, which generally reports a positive association between engagement and academic achievement. The difference should therefore be interpreted cautiously. One possible explanation is that the SEI combines attendance, discussion participation, and extracurricular involvement, which may not represent examination-focused study effort in the same way. Higher participation may also occur among students who require additional academic support or who face greater academic difficulty. In addition, attendance and discussion participation do not necessarily indicate the amount or quality of preparation for examinations, while extracurricular involvement may represent activities with limited direct correspondence to examination performance. These explanations are only possible interpretations and cannot be tested with the present cross sectional data. The unverified provenance of the dataset also limits confidence in assigning a substantive mechanism to the observed negative association.

Previous studies have reported associations between behavioral indicators, student engagement, and academic performance. Johar et al. (2023) reported that learning analytics provides information about student engagement patterns and their relationship with learning performance. Similarly, Al-Zahrani and Alasmari (2023) reported associations between data-driven educational interventions, instructional personalization, student engagement, and learning outcomes. These findings provide context for the present analysis but do not establish that the associations observed in the present dataset have the same direction or mechanism (Chung et al., 2020; Peng et al., 2026; Wang et al., 2022; Guo et al., 2024).

The present analysis does not provide empirical evidence of temporal memory or long term dynamic effects because the dataset contains no documented temporal ordering or repeated observations. The main contribution is therefore a transparent examination of statistical associations among learning behavior, student engagement, and academic performance, along with comparisons with structural and machine learning alternatives. The robustness results indicate that the principal associations remain relatively stable across the alternative static specifications, although overall predictive performance of the linear models remains limited.

From a practical perspective, the results provide descriptive and predictive information about educational indicators. However, the limited predictive performance of the baseline linear model and the cross-sectional nature of the data do not support recommendations based on causal or temporal effects. Future educational applications should combine these findings with longitudinal evidence and validated intervention studies before drawing conclusions about changes in student behavior or engagement.

## 4 Conclusion

The association between student engagement and academic performance is negative and statistically significant in the present dataset. After adjustment for learning behavior and the selected control variables, higher SEI values are associated with lower API values (γ_1_ = −1.2485, *p = 0.0014*). This direction differs from much of the previous educational literature, which generally reports a positive association between student engagement and academic achievement. The difference should therefore be interpreted cautiously. The present cross sectional analysis does not establish why higher SEI values correspond with lower API values in this dataset, and the observed association should not be interpreted as evidence of a causal relationship.

The baseline linear model shows limited explanatory performance, with *R*^2^ = 0.0035 and adjusted R^2^ = 0.0028. The corresponding RMSE is 17.665, MAE is 15.306, and MAPE is 24.17%. On the held out test data, the baseline model produces R^2^ = 0.006, RMSE = 17.696, MAE = 15.342, and MAPE = 24.108%. These results indicate limited out-of-sample predictive accuracy for the linear specification. Among the alternative predictive models, the random forest model provides stronger predictive performance than the baseline linear model.

The robustness analysis shows that the principal associations remain relatively stable across the alternative static specifications. Given the unverified provenance of the source dataset and the very limited explanatory power of the baseline linear model, the practical generalizability of these findings to educational policy is limited. The LBI-SEI association remains small and nonsignificant, whereas the SEI-API association remains negative and the LBI-API association remains positive in the specifications in which they are estimated. Model fit statistics show only limited variation across the robustness models.

The findings have limited practical interpretation because the data are cross-sectional and the analysis does not establish temporal ordering or causal mechanisms. The results therefore support identifying statistical associations and predictive patterns rather than claims that changes in learning behavior cause changes in engagement or that engagement causes changes in academic performance.

Several limitations should be considered. The analysis uses a publicly available Kaggle dataset whose original participant recruitment, institutional setting, sampling procedure, and collection protocol cannot be independently verified. The dataset therefore should not be presented as a verified sample of actual students. The raw dataset contained 1,534 exact duplicate records, which were removed before analysis. Several categorical variables are represented by numerical codes without complete publicly available coding documentation. In addition, the dataset contains no documented temporal variable or repeated longitudinal measurements. Consequently, these data cannot empirically evaluate temporal development or individual trajectories. The practical generalizability of the findings is limited. The dataset is publicly available through Kaggle, but its original participant recruitment, institutional setting, sampling procedure, and data collection protocol cannot be independently verified. In addition, the baseline linear model explains only a very small proportion of the variation in academic performance (R^2^ = 0.004). The findings should therefore be interpreted as dataset-specific statistical associations and predictive patterns rather than as evidence that can be directly generalized to educational policy or broader student populations.

Future research should use longitudinal educational datasets with documented participant provenance, repeated measurements, and explicit temporal identifiers. Such data would permit direct evaluation of temporal relationships and provide an appropriate basis for longitudinal modeling of changes in learning behavior, engagement, and academic performance.

## Data Availability

The original contributions presented in the study are included in the article/Supplementary material, further inquiries can be directed to the corresponding author.
