# Supplementary material for: Learning behavior, student engagement, and academic performance in learning analytics
Source: Front Psychol. 2026 Sep 14;17:1908197. doi: 10.3389/fpsyg.2026.1908197 (PMC13617089; doi:10.3389/fpsyg.2026.1908197)
Supplement: Supplementary file 1 [file Supplementary_File_1.docx]

**Table S1.** Software, Packages, and Model Implementation Settings

| Analysis / Model | Software / Package | Version | Main settings |
| --- | --- | --- | --- |
| Data processing | Python / pandas | 3.14.7 / 3.0.5 | Data preparation and preprocessing |
| Numerical computation | NumPy / SciPy | 2.5.2 / 1.18.1 | Numerical and statistical calculations |
| OLS regression | statsmodels | 0.14.6 | Ordinary least squares |
| Observed variable path model | semopy | 2.3.11 | Observed composite variables |
| Random forest regression | scikit-learn | 1.9.0 | 100 trees; maximum depth = None; minimum split = 2; minimum leaf = 1; bootstrap = True |
| Gradient boosting regression | scikit-learn | 1.9.0 | 100 estimators; learning rate = 0.10; maximum depth = 3; minimum split = 2; minimum leaf = 1 |
| Support vector regression | scikit-learn | 1.9.0 | RBF kernel; C = 1.0; epsilon = 0.10; gamma = scale |
| Train-test splitting | scikit-learn | 1.9.0 | 80% training; 20% test; random seed = 42 |
| Predictor standardization | scikit-learn | 1.9.0 | Training data only |
| Bootstrap validation | Python / SciPy | 3.14.7 / 1.18.1 | Nonparametric bootstrap; 95% percentile confidence intervals |
