# Supplementary material for: Learning behavior, student engagement, and academic performance in learning analytics
Source: Front Psychol. 2026 Sep 14;17:1908197. doi: 10.3389/fpsyg.2026.1908197 (PMC13617089; doi:10.3389/fpsyg.2026.1908197)
Supplement: Supplementary file 2 [file Supplementary_File_2.docx]

\# ============================================================

\# FULL REPRODUCIBILITY CODE

\# Cross-sectional educational analytics analysis

\# ============================================================

\# ============================================================

\# 1. IMPORT PACKAGES

\# ============================================================

import os

import numpy as np

import pandas as pd

import statsmodels.api as sm

import statsmodels.formula.api as smf

from scipy import stats

from sklearn.preprocessing import StandardScaler

from sklearn.model_selection import train_test_split

import matplotlib.pyplot as plt

\# ============================================================

\# 2. FILE PATH

\# ============================================================

\# Change this path to the location of the CSV file.

FILE_PATH = "student_performance.csv"

\# Folder for output files

OUTPUT_DIR = "analysis_outputs"

os.makedirs(OUTPUT_DIR, exist_ok=True)

\# ============================================================

\# 3. READ THE DATASET

\# ============================================================

df_raw = pd.read_csv(FILE_PATH)

print("Raw dataset dimensions:")

print("Rows:", df_raw.shape[0])

print("Columns:", df_raw.shape[1])

print("\nColumn names:")

print(df_raw.columns.tolist())

\# ============================================================

\# 4. DEFINE REQUIRED VARIABLES

\# ============================================================

required_columns = [

"StudyHours",

"Attendance",

"Resources",

"Extracurricular",

"Motivation",

"Internet",

"Gender",

"Age",

"LearningStyle",

"OnlineCourses",

"Discussions",

"AssignmentCompletion",

"ExamScore",

"EduTech",

"StressLevel",

"FinalGrade"

]

\# ============================================================

\# 5. CHECK THAT ALL VARIABLES ARE PRESENT

\# ============================================================

missing_columns = [

col for col in required_columns

if col not in df_raw.columns

]

if len(missing_columns) > 0:

raise ValueError(

"The following required variables are missing: "

+ str(missing_columns)

)

print("\nAll 16 required variables are present.")

\# ============================================================

\# 6. KEEP ONLY THE REQUIRED 16 VARIABLES

\# ============================================================

df = df_raw[required_columns].copy()

\# ============================================================

\# 7. CONVERT VARIABLES TO NUMERIC

\# ============================================================

for col in required_columns:

df[col] = pd.to_numeric(df[col], errors="coerce")

\# ============================================================

\# 8. CHECK DATA TYPES

\# ============================================================

print("\nData types:")

print(df.dtypes)

\# ============================================================

\# 9. CHECK MISSING VALUES BEFORE CLEANING

\# ============================================================

missing_before = df.isna().sum()

print("\nMissing values by variable:")

print(missing_before)

total_missing = int(missing_before.sum())

print("\nTotal missing values:", total_missing)

\# ============================================================

\# 10. CHECK EXACT DUPLICATES

\# ============================================================

duplicate_mask = df.duplicated(

subset=required_columns,

keep="first"

)

duplicate_count = int(duplicate_mask.sum())

print("\nExact duplicate records:", duplicate_count)

\# ============================================================

\# 11. REMOVE EXACT DUPLICATES

\# ============================================================

df_clean = df.loc[~duplicate_mask].copy()

print("\nRecords after duplicate removal:")

print(len(df_clean))

\# ============================================================

\# 12. CHECK MISSING VALUES AFTER DUPLICATE REMOVAL

\# ============================================================

missing_after = df_clean.isna().sum()

print("\nMissing values after duplicate removal:")

print(missing_after)

if missing_after.sum() > 0:

raise ValueError(

"Missing values remain in the analytical dataset."

)

\# ============================================================

\# 13. DEFINE EXPECTED VARIABLE RANGES

\# ============================================================

valid_ranges = {

"StudyHours": (0, 44),

"Attendance": (60, 100),

"Resources": (0, 2),

"Extracurricular": (0, 1),

"Motivation": (0, 2),

"Internet": (0, 1),

"Gender": (0, 1),

"Age": (18, 29),

"LearningStyle": (0, 3),

"OnlineCourses": (0, 20),

"Discussions": (0, 1),

"AssignmentCompletion": (50, 100),

"ExamScore": (40, 100),

"EduTech": (0, 1),

"StressLevel": (0, 2),

"FinalGrade": (0, 3)

}

\# ============================================================

\# 14. CHECK INVALID VALUES

\# ============================================================

invalid_records = {}

for variable, (lower, upper) in valid_ranges.items():

mask = (

(df_clean[variable] < lower)

|

(df_clean[variable] > upper)

)

count = int(mask.sum())

invalid_records[variable] = count

print(

f"{variable}: "

f"{count} observations outside the expected range"

)

total_invalid = sum(invalid_records.values())

print("\nTotal invalid observations detected:", total_invalid)

\# ============================================================

\# 15. STOP IF INVALID VALUES ARE FOUND

\# ============================================================

\# The manuscript states that no invalid values were found.

\# Therefore, the code stops if invalid values are detected

\# rather than silently deleting them.

if total_invalid > 0:

raise ValueError(

"Invalid values were detected. "

"Review the records before continuing."

)

\# ============================================================

\# 16. FINAL ANALYTICAL DATASET

\# ============================================================

N = len(df_clean)

print("\nFinal analytical sample size:", N)

if N != 12469:

print(

"WARNING: The current dataset does not contain "

"12,469 records after cleaning."

)

else:

print(

"Confirmed: 12,469 unique records remain."

)

\# ============================================================

\# 17. SAVE CLEAN DATASET

\# ============================================================

df_clean.to_csv(

os.path.join(

OUTPUT_DIR,

"cleaned_dataset.csv"

),

index=False

)

\# ============================================================

\# 18. DEFINE INDEX VARIABLES

\# ============================================================

learning_variables = [

"StudyHours",

"OnlineCourses",

"AssignmentCompletion",

"LearningStyle"

]

engagement_variables = [

"Attendance",

"Discussions",

"AssignmentCompletion",

"Extracurricular"

]

performance_variables = [

"ExamScore",

"FinalGrade"

]

\# ============================================================

\# 19. STANDARDIZE CONTINUOUS INDEX VARIABLES

\# ============================================================

\# The manuscript states that continuous variables are

\# standardized before index construction.

continuous_index_variables = [

"StudyHours",

"OnlineCourses",

"AssignmentCompletion",

"Attendance",

"ExamScore",

"FinalGrade"

]

scaler = StandardScaler()

df_clean[

continuous_index_variables

] = scaler.fit_transform(

df_clean[

continuous_index_variables

]

)

\# ============================================================

\# 20. HANDLE LEARNING STYLE

\# ============================================================

\# LearningStyle is coded as a categorical variable in the

\# manuscript. It is therefore NOT automatically standardized

\# as a continuous measurement.

\# If the original analysis treated LearningStyle numerically,

\# retain the supplied numerical code.

df_clean["LearningStyle_Index"] = (

df_clean["LearningStyle"]

)

\# ============================================================

\# 21. DEFINE INDEX WEIGHTS

\# ============================================================

\# IMPORTANT:

\# The manuscript defines wj, vk, and rl but does not provide

\# their numerical values.

\#

\# The following code uses equal weights.

\#

\# Replace these values if the actual analysis used different

\# weights.

w = {

"StudyHours": 1 / 4,

"OnlineCourses": 1 / 4,

"AssignmentCompletion": 1 / 4,

"LearningStyle_Index": 1 / 4

}

v = {

"Attendance": 1 / 4,

"Discussions": 1 / 4,

"AssignmentCompletion": 1 / 4,

"Extracurricular": 1 / 4

}

r = {

"ExamScore": 1 / 2,

"FinalGrade": 1 / 2

}

\# ============================================================

\# 22. CONSTRUCT LEARNING BEHAVIOR INDEX

\# ============================================================

df_clean["LBI"] = (

w["StudyHours"]

* df_clean["StudyHours"]

+

w["OnlineCourses"]

* df_clean["OnlineCourses"]

+

w["AssignmentCompletion"]

* df_clean["AssignmentCompletion"]

+

w["LearningStyle_Index"]

* df_clean["LearningStyle_Index"]

)

\# ============================================================

\# 23. CONSTRUCT STUDENT ENGAGEMENT INDEX

\# ============================================================

df_clean["SEI"] = (

v["Attendance"]

* df_clean["Attendance"]

+

v["Discussions"]

* df_clean["Discussions"]

+

v["AssignmentCompletion"]

* df_clean["AssignmentCompletion"]

+

v["Extracurricular"]

* df_clean["Extracurricular"]

)

\# ============================================================

\# 24. CONSTRUCT ACADEMIC PERFORMANCE INDEX

\# ============================================================

df_clean["API"] = (

r["ExamScore"]

* df_clean["ExamScore"]

+

r["FinalGrade"]

* df_clean["FinalGrade"]

)

\# ============================================================

\# 25. CHECK INDEX VALUES

\# ============================================================

print("\nIndex summary:")

print(

df_clean[

["LBI", "SEI", "API"]

].describe()

)

\# ============================================================

\# 26. SAVE DATASET WITH INDICES

\# ============================================================

df_clean.to_csv(

os.path.join(

OUTPUT_DIR,

"analytical_dataset_with_indices.csv"

),

index=False

)

\# ============================================================

\# 27. DEFINE CONTROL VARIABLES

\# ============================================================

control_variables = [

"Age",

"Gender",

"Motivation",

"StressLevel",

"EduTech",

"Internet",

"Resources"

]

\# ============================================================

\# 28. STANDARDIZE CONTROL VARIABLES WHERE REQUIRED

\# ============================================================

\# Age is continuous.

\# Other variables retain their supplied numerical coding.

control_scaler = StandardScaler()

df_clean["Age_std"] = control_scaler.fit_transform(

df_clean[["Age"]]

)

\# ============================================================

\# 29. MODEL 1

\# LEARNING BEHAVIOR -> STUDENT ENGAGEMENT

\# ============================================================

X1 = df_clean[

[

"LBI",

"Age_std",

"Gender",

"Motivation",

"StressLevel",

"EduTech",

"Internet",

"Resources"

]

]

X1 = sm.add_constant(X1)

y1 = df_clean["SEI"]

\# ============================================================

\# 30. ESTIMATE MODEL 1 USING OLS

\# ============================================================

model1 = sm.OLS(

y1,

X1

).fit()

\# ============================================================

\# 31. DISPLAY MODEL 1

\# ============================================================

print("\n==============================")

print("MODEL 1: SEI")

print("==============================")

print(

model1.summary()

)

\# ============================================================

\# 32. MODEL 2

\# STUDENT ENGAGEMENT + LEARNING BEHAVIOR -> API

\# ============================================================

X2 = df_clean[

[

"SEI",

"LBI",

"Age_std",

"Gender",

"Motivation",

"StressLevel",

"EduTech",

"Internet",

"Resources"

]

]

X2 = sm.add_constant(X2)

y2 = df_clean["API"]

\# ============================================================

\# 33. ESTIMATE MODEL 2 USING OLS

\# ============================================================

model2 = sm.OLS(

y2,

X2

).fit()

\# ============================================================

\# 34. DISPLAY MODEL 2

\# ============================================================

print("\n==============================")

print("MODEL 2: API")

print("==============================")

print(

model2.summary()

)

\# ============================================================

\# 35. CREATE PREDICTIONS

\# ============================================================

df_clean["SEI_predicted"] = (

model1.predict(X1)

)

df_clean["API_predicted"] = (

model2.predict(X2)

)

\# ============================================================

\# 36. CALCULATE RESIDUALS

\# ============================================================

df_clean["SEI_residual"] = (

df_clean["SEI"]

-

df_clean["SEI_predicted"]

)

df_clean["API_residual"] = (

df_clean["API"]

-

df_clean["API_predicted"]

)

\# ============================================================

\# 37. RESIDUAL SUM OF SQUARES

\# ============================================================

RSS_SEI = np.sum(

df_clean["SEI_residual"] ** 2

)

RSS_API = np.sum(

df_clean["API_residual"] ** 2

)

print("\nRSS for SEI model:", RSS_SEI)

print(

"RSS for API model:",

RSS_API

)

\# ============================================================

\# 38. RESIDUAL VARIANCE

\# ============================================================

sigma2_SEI = (

RSS_SEI

/

model1.df_resid

)

sigma2_API = (

RSS_API

/

model2.df_resid

)

print(

"\nResidual variance SEI:",

sigma2_SEI

)

print(

"Residual variance API:",

sigma2_API

)

\# ============================================================

\# 39. STANDARD ERRORS

\# ============================================================

SE_model1 = model1.bse

SE_model2 = model2.bse

print("\nStandard errors, Model 1:")

print(SE_model1)

print("\nStandard errors, Model 2:")

print(SE_model2)

\# ============================================================

\# 40. T STATISTICS

\# ============================================================

t_model1 = model1.tvalues

t_model2 = model2.tvalues

print("\nT statistics, Model 1:")

print(t_model1)

print("\nT statistics, Model 2:")

print(t_model2)

\# ============================================================

\# 41. P VALUES

\# ============================================================

p_model1 = model1.pvalues

p_model2 = model2.pvalues

print("\nP values, Model 1:")

print(p_model1)

print("\nP values, Model 2:")

print(p_model2)

\# ============================================================

\# 42. CONFIDENCE INTERVALS

\# ============================================================

CI_model1 = model1.conf_int(

alpha=0.05

)

CI_model2 = model2.conf_int(

alpha=0.05

)

CI_model1.columns = [

"CI_lower",

"CI_upper"

]

CI_model2.columns = [

"CI_lower",

"CI_upper"

]

print("\n95% confidence intervals, Model 1:")

print(CI_model1)

print("\n95% confidence intervals, Model 2:")

print(CI_model2)

\# ============================================================

\# 43. R-SQUARED

\# ============================================================

R2_SEI = model1.rsquared

R2_API = model2.rsquared

print("\nR² SEI model:", R2_SEI)

print("R² API model:", R2_API)

\# ============================================================

\# 44. ADJUSTED R-SQUARED

\# ============================================================

AdjR2_SEI = model1.rsquared_adj

AdjR2_API = model2.rsquared_adj

print(

"\nAdjusted R² SEI model:",

AdjR2_SEI

)

print(

"Adjusted R² API model:",

AdjR2_API

)

\# ============================================================

\# 45. RMSE

\# ============================================================

RMSE_SEI = np.sqrt(

np.mean(

df_clean["SEI_residual"] ** 2

)

)

RMSE_API = np.sqrt(

np.mean(

df_clean["API_residual"] ** 2

)

)

print("\nRMSE SEI:", RMSE_SEI)

print("RMSE API:", RMSE_API)

\# ============================================================

\# 46. MAE

\# ============================================================

MAE_SEI = np.mean(

np.abs(

df_clean["SEI_residual"]

)

)

MAE_API = np.mean(

np.abs(

df_clean["API_residual"]

)

)

print("\nMAE SEI:", MAE_SEI)

print("MAE API:", MAE_API)

\# ============================================================

\# 47. MAPE

\# ============================================================

def calculate_mape(

actual,

predicted

):

actual = np.asarray(actual)

predicted = np.asarray(predicted)

nonzero = actual != 0

return (

100

*

np.mean(

np.abs(

(

actual[nonzero]

-

predicted[nonzero]

)

/

actual[nonzero]

)

)

)

MAPE_SEI = calculate_mape(

df_clean["SEI"],

df_clean["SEI_predicted"]

)

MAPE_API = calculate_mape(

df_clean["API"],

df_clean["API_predicted"]

)

print("\nMAPE SEI:", MAPE_SEI)

print("MAPE API:", MAPE_API)

\# ============================================================

\# 48. MODEL PERFORMANCE SUMMARY

\# ============================================================

performance_summary = pd.DataFrame({

"Model": [

"Student Engagement Model",

"Academic Performance Model"

],

"N": [

int(model1.nobs),

int(model2.nobs)

],

"R2": [

R2_SEI,

R2_API

],

"Adjusted_R2": [

AdjR2_SEI,

AdjR2_API

],

"RMSE": [

RMSE_SEI,

RMSE_API

],

"MAE": [

MAE_SEI,

MAE_API

],

"MAPE_percent": [

MAPE_SEI,

MAPE_API

]

})

print("\nModel performance:")

print(performance_summary)

\# ============================================================

\# 49. SAVE MODEL PERFORMANCE

\# ============================================================

performance_summary.to_csv(

os.path.join(

OUTPUT_DIR,

"model_performance.csv"

),

index=False

)

\# ============================================================

\# 50. COEFFICIENT TABLE FUNCTION

\# ============================================================

def coefficient_table(model):

table = pd.DataFrame({

"Coefficient": model.params,

"Standard_Error": model.bse,

"t_value": model.tvalues,

"p_value": model.pvalues

})

ci = model.conf_int(

alpha=0.05

)

table["CI_lower"] = ci[0]

table["CI_upper"] = ci[1]

return table

\# ============================================================

\# 51. COEFFICIENT TABLES

\# ============================================================

coef_model1 = coefficient_table(

model1

)

coef_model2 = coefficient_table(

model2

)

print("\nModel 1 coefficients:")

print(coef_model1)

print("\nModel 2 coefficients:")

print(coef_model2)

\# ============================================================

\# 52. SAVE COEFFICIENT TABLES

\# ============================================================

coef_model1.to_csv(

os.path.join(

OUTPUT_DIR,

"model1_coefficients.csv"

)

)

coef_model2.to_csv(

os.path.join(

OUTPUT_DIR,

"model2_coefficients.csv"

)

)

\# ============================================================

\# 53. RESIDUAL DIAGNOSTICS

\# ============================================================

def residual_diagnostics(

residuals,

model_name

):

residuals = np.asarray(

residuals

)

diagnostics = {

"Mean_residual":

np.mean(residuals),

"Residual_SD":

np.std(

residuals,

ddof=1

),

"Minimum_residual":

np.min(residuals),

"Maximum_residual":

np.max(residuals),

"Skewness":

stats.skew(residuals),

"Kurtosis":

stats.kurtosis(

residuals,

fisher=False

)

}

result = pd.DataFrame(

[diagnostics]

)

result.insert(

0,

"Model",

model_name

)

return result

diag_model1 = residual_diagnostics(

df_clean["SEI_residual"],

"Student Engagement Model"

)

diag_model2 = residual_diagnostics(

df_clean["API_residual"],

"Academic Performance Model"

)

\# ============================================================

\# 54. COMBINE DIAGNOSTICS

\# ============================================================

diagnostics = pd.concat(

[

diag_model1,

diag_model2

],

ignore_index=True

)

print("\nResidual diagnostics:")

print(diagnostics)

\# ============================================================

\# 55. SAVE DIAGNOSTICS

\# ============================================================

diagnostics.to_csv(

os.path.join(

OUTPUT_DIR,

"residual_diagnostics.csv"

),

index=False

)

\# ============================================================

\# 56. RESIDUAL HISTOGRAM: SEI

\# ============================================================

plt.figure(

figsize=(8, 5)

)

plt.hist(

df_clean["SEI_residual"],

bins=30

)

plt.xlabel(

"Residual"

)

plt.ylabel(

"Frequency"

)

plt.title(

"Residual Distribution: Student Engagement Model"

)

plt.tight_layout()

plt.savefig(

os.path.join(

OUTPUT_DIR,

"SEI_residual_distribution.png"

),

dpi=300

)

plt.close()

\# ============================================================

\# 57. RESIDUAL HISTOGRAM: API

\# ============================================================

plt.figure(

figsize=(8, 5)

)

plt.hist(

df_clean["API_residual"],

bins=30

)

plt.xlabel(

"Residual"

)

plt.ylabel(

"Frequency"

)

plt.title(

"Residual Distribution: Academic Performance Model"

)

plt.tight_layout()

plt.savefig(

os.path.join(

OUTPUT_DIR,

"API_residual_distribution.png"

),

dpi=300

)

plt.close()

\# ============================================================

\# 58. RESIDUALS VS FITTED: SEI

\# ============================================================

plt.figure(

figsize=(8, 5)

)

plt.scatter(

df_clean["SEI_predicted"],

df_clean["SEI_residual"],

alpha=0.5

)

plt.axhline(

y=0,

linestyle="--"

)

plt.xlabel(

"Fitted SEI"

)

plt.ylabel(

"Residual"

)

plt.title(

"Residuals versus Fitted Values: SEI Model"

)

plt.tight_layout()

plt.savefig(

os.path.join(

OUTPUT_DIR,

"SEI_residuals_vs_fitted.png"

),

dpi=300

)

plt.close()

\# ============================================================

\# 59. RESIDUALS VS FITTED: API

\# ============================================================

plt.figure(

figsize=(8, 5)

)

plt.scatter(

df_clean["API_predicted"],

df_clean["API_residual"],

alpha=0.5

)

plt.axhline(

y=0,

linestyle="--"

)

plt.xlabel(

"Fitted API"

)

plt.ylabel(

"Residual"

)

plt.title(

"Residuals versus Fitted Values: API Model"

)

plt.tight_layout()

plt.savefig(

os.path.join(

OUTPUT_DIR,

"API_residuals_vs_fitted.png"

),

dpi=300

)

plt.close()

\# ============================================================

\# 60. ROBUST STANDARD ERRORS

\# ============================================================

\# HC3 heteroscedasticity-consistent covariance estimator

model1_HC3 = model1.get_robustcov_results(

cov_type="HC3"

)

model2_HC3 = model2.get_robustcov_results(

cov_type="HC3"

)

\# ============================================================

\# 61. ROBUST COEFFICIENT TABLES

\# ============================================================

def robust_coefficient_table(model):

parameters = model.params

standard_errors = model.bse

t_values = model.tvalues

p_values = model.pvalues

confidence_intervals = model.conf_int()

table = pd.DataFrame({

"Coefficient":

parameters,

"Robust_SE":

standard_errors,

"t_value":

t_values,

"p_value":

p_values,

"CI_lower":

confidence_intervals[:, 0],

"CI_upper":

confidence_intervals[:, 1]

})

return table

robust_table1 = robust_coefficient_table(

model1_HC3

)

robust_table2 = robust_coefficient_table(

model2_HC3

)

\# ============================================================

\# 62. SAVE ROBUST STANDARD ERROR RESULTS

\# ============================================================

robust_table1.to_csv(

os.path.join(

OUTPUT_DIR,

"model1_HC3_results.csv"

)

)

robust_table2.to_csv(

os.path.join(

OUTPUT_DIR,

"model2_HC3_results.csv"

)

)

\# ============================================================

\# 63. ALTERNATIVE STATIC SPECIFICATION 1

\# WITHOUT CONTROL VARIABLES

\# ============================================================

X1_alt1 = sm.add_constant(

df_clean[

["LBI"]

]

)

alt_model1 = sm.OLS(

df_clean["SEI"],

X1_alt1

).fit()

\# ============================================================

\# 64. ALTERNATIVE STATIC SPECIFICATION 2

\# WITHOUT CONTROL VARIABLES

\# ============================================================

X2_alt1 = sm.add_constant(

df_clean[

["SEI", "LBI"]

]

)

alt_model2 = sm.OLS(

df_clean["API"],

X2_alt1

).fit()

\# ============================================================

\# 65. ALTERNATIVE SPECIFICATION 3

\# API WITH SEI ONLY

\# ============================================================

X2_alt2 = sm.add_constant(

df_clean[

["SEI"]

]

)

alt_model3 = sm.OLS(

df_clean["API"],

X2_alt2

).fit()

\# ============================================================

\# 66. ALTERNATIVE SPECIFICATION 4

\# API WITH LBI ONLY

\# ============================================================

X2_alt3 = sm.add_constant(

df_clean[

["LBI"]

]

)

alt_model4 = sm.OLS(

df_clean["API"],

X2_alt3

).fit()

\# ============================================================

\# 67. ROBUSTNESS SUMMARY

\# ============================================================

robustness_summary = pd.DataFrame({

"Specification": [

"Baseline SEI",

"SEI without controls",

"Baseline API",

"API without controls",

"API with SEI only",

"API with LBI only"

],

"R2": [

model1.rsquared,

alt_model1.rsquared,

model2.rsquared,

alt_model2.rsquared,

alt_model3.rsquared,

alt_model4.rsquared

],

"Adjusted_R2": [

model1.rsquared_adj,

alt_model1.rsquared_adj,

model2.rsquared_adj,

alt_model2.rsquared_adj,

alt_model3.rsquared_adj,

alt_model4.rsquared_adj

],

"RMSE": [

np.sqrt(

np.mean(

model1.resid ** 2

)

),

np.sqrt(

np.mean(

alt_model1.resid ** 2

)

),

np.sqrt(

np.mean(

model2.resid ** 2

)

),

np.sqrt(

np.mean(

alt_model2.resid ** 2

)

),

np.sqrt(

np.mean(

alt_model3.resid ** 2

)

),

np.sqrt(

np.mean(

alt_model4.resid ** 2

)

)

],

"MAE": [

np.mean(

np.abs(

model1.resid

)

),

np.mean(

np.abs(

alt_model1.resid

)

),

np.mean(

np.abs(

model2.resid

)

),

np.mean(

np.abs(

alt_model2.resid

)

),

np.mean(

np.abs(

alt_model3.resid

)

),

np.mean(

np.abs(

alt_model4.resid

)

)

]

})

print(

"\n=============================="

)

print(

"ROBUSTNESS ANALYSIS"

)

print(

"=============================="

)

print(

robustness_summary

)

\# ============================================================

\# 68. SAVE ROBUSTNESS RESULTS

\# ============================================================

robustness_summary.to_csv(

os.path.join(

OUTPUT_DIR,

"robustness_results.csv"

),

index=False

)

\# ============================================================

\# 69. EXPORT COMPLETE RESULTS TO EXCEL

\# ============================================================

excel_path = os.path.join(

OUTPUT_DIR,

"complete_analysis_results.xlsx"

)

with pd.ExcelWriter(

excel_path,

engine="openpyxl"

) as writer:

performance_summary.to_excel(

writer,

sheet_name="Model_Performance",

index=False

)

coef_model1.to_excel(

writer,

sheet_name="SEI_Coefficients"

)

coef_model2.to_excel(

writer,

sheet_name="API_Coefficients"

)

diagnostics.to_excel(

writer,

sheet_name="Residual_Diagnostics",

index=False

)

robustness_summary.to_excel(

writer,

sheet_name="Robustness",

index=False

)

\# ============================================================

\# 70. SAVE FINAL ANALYTICAL DATASET

\# ============================================================

df_clean.to_csv(

os.path.join(

OUTPUT_DIR,

"FINAL_ANALYTICAL_DATASET.csv"

),

index=False

)

\# ============================================================

\# 71. FINAL SUMMARY

\# ============================================================

print("\n")

print("=" * 60)

print("ANALYSIS COMPLETED")

print("=" * 60)

print(

"Raw records:",

len(df_raw)

)

print(

"Exact duplicates removed:",

duplicate_count

)

print(

"Final analytical records:",

len(df_clean)

)

print(

"Number of variables:",

len(required_columns)

)

print(

"Missing values:",

int(df_clean[required_columns].isna().sum().sum())

)

print(

"SEI model R²:",

model1.rsquared

)

print(

"API model R²:",

model2.rsquared

)

print(

"SEI model RMSE:",

RMSE_SEI

)

print(

"API model RMSE:",

RMSE_API

)

print(

"SEI model MAE:",

MAE_SEI

)

print(

"API model MAE:",

MAE_API

)

print(

"SEI model MAPE:",

MAPE_SEI

)

print(

"API model MAPE:",

MAPE_API

)

print(

"\nResults saved to:",

OUTPUT_DIR

)

print(

"\nExcel results file:",

excel_path

)
